# When Cytokinin, a Plant Hormone, Meets the Adenosine A_2A_ Receptor: A Novel Neuroprotectant and Lead for Treating Neurodegenerative Disorders?

**DOI:** 10.1371/journal.pone.0038865

**Published:** 2012-06-18

**Authors:** Yi-Chao Lee, Ying-Chen Yang, Chuen-Lin Huang, Tsun-Yung Kuo, Jung-Hsin Lin, De-Ming Yang, Nai-Kuei Huang

**Affiliations:** 1 Ph.D. Program for Neural Regenerative Medicine, College of Medical Science and Technology, Taipei Medical University, Taipei, Taiwan, Republic of China; 2 Department of Animal Science, National Ilan University, Ilan, Taiwan, Republic of China; 3 Medical Research Center, Cardinal Tien Hospital, Hsintien, New Taipei City, Taiwan, Republic of China; 4 Graduate Institute of Physiology and Department of Physiology and Biophysics, National Defense Medical Center, Taipei, Taiwan, Republic of China; 5 Institute of Biotechnology, National Ilan University, Ilan, Taiwan, Republic of China; 6 School of Pharmacy, National Taiwan University, Taipei, Taiwan, Republic of China; 7 Division of Mechanics, Research Center for Applied Sciences, Academia Sinica, Taipei, Taiwan, Republic of China; 8 Institute of Biomedical Sciences, Academia Sinica, Nankang, Taipei, Taiwan, Republic of China; 9 Department of Medical Research and Education, Taipei Veterans General Hospital, Taipei, Taiwan, Republic of China; 10 Institute of Biophotonics, National Yang-Ming University, Taipei, Taiwan, Republic of China; 11 National Research Institute of Chinese Medicine, Taipei, Taiwan, Republic of China; Ohio State University, United States of America

## Abstract

It is well known that cytokinins are a class of phytohormones that promote cell division in plant roots and shoots. However, their targets, biological functions, and implications in mammalian systems have rarely been examined. In this study, we show that one cytokinin, zeatin riboside, can prevent pheochromocytoma (PC12) cells from serum deprivation-induced apoptosis by acting on the adenosine A_2A_ receptor (A_2A_-R), which was blocked by an A_2A_-R antagonist and a protein kinase A (PKA) inhibitor, demonstrating the functional ability of zeatin riboside by mediating through A_2A_-R signaling event. Since the A_2A_-R was implicated as a therapeutic target in treating Huntington’s disease (HD), a cellular model of HD was applied by transfecting mutant huntingtin in PC12 cells. By using filter retardation assay and confocal microscopy we found that zeatin riboside reversed mutant huntingtin (Htt)-induced protein aggregations and proteasome deactivation through A_2A_-R signaling. PKA inhibitor blocked zeatin riboside-induced suppression of mutant Htt aggregations. In addition, PKA activated proteasome activity and reduced mutant Htt protein aggregations. However, a proteasome inhibitor blocked both zeatin riboside-and PKA activator-mediated suppression of mutant Htt aggregations, confirming mediation of the A_2A_-R/PKA/proteasome pathway. Taken together, zeatin riboside might have therapeutic potential as a novel neuroprotectant and a lead for treating neurodegenerative disorders.

## Introduction

Cytokinins are plant hormones that play essential roles in regulating various aspects of plant growth and development, such as *de novo* bud formation, release of buds from apical dominance, leaf expansion, chloroplast formation, delay of senescence, promotion of seed germination, and control of the cell cycle [1], [2]. Naturally occurring cytokinins are mainly adenine derivatives, such as isopentyladenine and *trans*-zeatin, and synthetic cytokinins include some adenine analogues, such as 6-benzyladenine and kinetin. In plants, the cytokinin signaling pathway is similar to bacterial and yeast two-component signal transduction pathways; it is specifically similar to histidine-aspartate multi-step phosphorelays, which are comprised of sensor kinases, histidine phosphotransfer proteins, and response regulators [3]. In animal cells, cytokinins are also of interest for their antioxidative, antitumorigenic, and anti-aging activities [4]–[7].

Previously, cytokinin-binding proteins were found in mammalian sera [8], demonstrating the existence of mammalian targeting proteins for plant hormones. Later, Froldi et al. [9] showed that 6-benzyladenine could act on a purinergic type-2 receptor that induces calcium mobilization in rat atria, suggesting a membrane-bound protein for cytokinins in animal cells. In addition, since cytokinins are important at specific phases of the plant cell cycle [10], in parallel, their anticancer effects were attributed to mediation by cyclins [11] or cyclin-dependent kinases [12], [13], suggesting the potential for cytokinins as anticancer drugs [7]. Thus, cytokinin might be used as a plant hormone and also could possibly serve as a candidate for treating human diseases. However, most of the targeting sites and mechanisms are still mostly unknown.

On the other hand, purinoreceptors can be subdivided into P1 receptors, which bind adenosine as a natural ligand, and P2 receptors, which bind ATP, ADP, and adenine dinucleotides, but also pyrimidines like UTP and UDP [14]. On the basis of cloning, pharmacology, and transduction mechanisms, the P1 receptor family is divided into four subtypes (A_1_, A_2A_, A_2B_, and A_3_), while the P2 receptor family is divided into P2X ionotropic receptors (P2X_1–7_) and P2Y metabotropic G protein-coupled receptors (P2Y_1_, _2_, _4_, _6_, and _11–14_) [15]. These receptors are involved in regulating health and disease [16], including neuroprotection and neurodegeneration [17]–[20], such as ischemia, epilepsy, depression, Alzheimer’s disease (AD), Parkinson’s disease (PD), and Huntington's disease (HD). Therefore, due to the close connections of these purinoreceptors in regulating diverse physiological and pathological neuronal functions, recent advances in therapies using purinergic-related drugs in a wide range of pathological conditions have occurred [18], [21]–[23]. In addition, 6-benzyladenine can act on the P2 receptor [9], and almost all cytokinins are present in plants as both a free base and corresponding nucleosides and nucleotides [24] which have similar adenosine-based structures as agonists of P1 receptors. We thus questioned if cytokinins (with or without ribosides) can act on P1 receptors. Among them, the adenosine A_2A_ receptor (A_2A_-R) has drawn attention as a potential therapeutic drug target in HD because it is highly prevalent in the striatum where mutant huntingtin (Htt) causes selective neural cell loss and atrophy. Therefore, A_2A_-R-related drugs were suggested for treating HD [25], [26].

HD is an autosomal dominant neurodegenerative disorder caused by the expansion of a glutamine repeat in the Htt with a distinct phenotype characterized by chorea, dystonia, incoordination, cognitive decline, and behavioral difficulties [27]. Mutant Htt results from a CAG trinucleotide expansion in exon 1 leading to an expanded polyglutamine (polyQ) strand at the N terminus and a putative toxic gain of function. Normally, the *Htt* gene has 35 or fewer CAG repeats in its N-terminal region, whereas that of HD patients is associated with 36 or more repeats. The numbers of CAG repeats is negatively correlated with the onset age of HD [28]. During disease progression, concentration and short-term memory diminish, and involuntary movements of the head, trunk, and limbs increase. Finally, death results from complications such as choking, infection, and heart failure. Currently, therapeutic strategies for treating HD patients are mostly for symptom relief, and some treatments have unfavorable side effects [29]. Therapeutic drugs to treat HD are urgently needed to be developed.

Collectively, in this study, we found that cytokinin can act on the A_2A_-R and prevent mutant Htt aggregations suggesting that cytokinin could possibly be applied as a lead or a novel neuroprotectant for treating neurodegenerative disorders.

## Results

### Zeatin Riboside Activates the A_2A_-R Signaling and Prevents Serum Deprivation-induced Apoptosis

**Figure 1:**
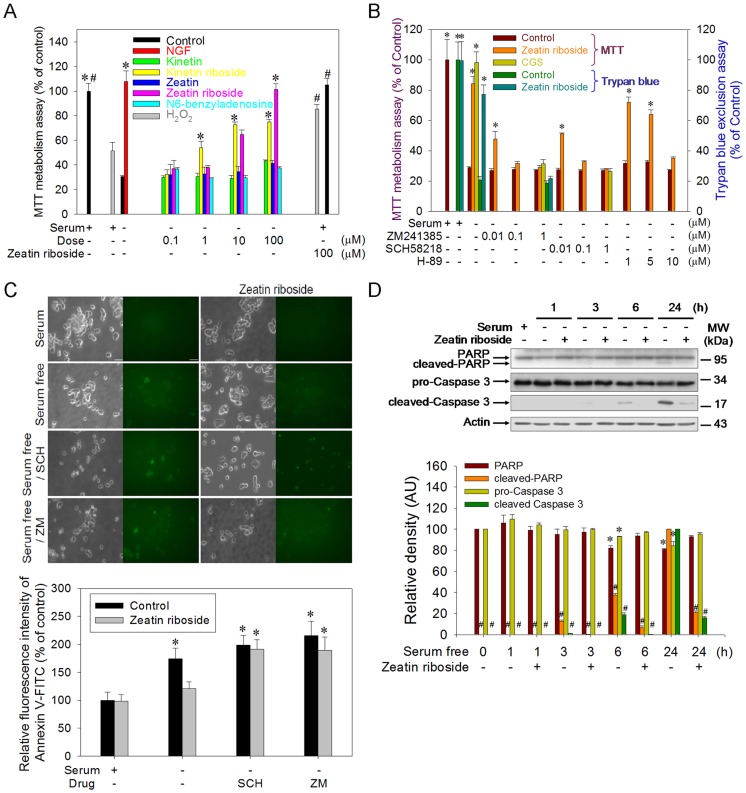
Cytokinins acting on the A_2A_-R prevent serum deprivation-induced PC12 cell apoptosis. (A) Serum-contained and serum-deprived cells were treated with or without the indicated reagent(s) for 24 h. NGF were treated in 100 ng/ml. Besides, cells pretreated with zeatin riboside (100 µM) for 3 h were treated with or without H_2_O_2_ (25 µM) for 24 h. Cell viability was expressed as a percentage of the results from the MTT assay with respect to the mean value of the serum-contained control group. Data points represent the mean ± SEM (*n* = 3∼6). *p<0.05, compared to its serum-deprived group. #*p*<0.05, compared to the H_2_O_2_-treated group. (B) Serum-deprived cells were pretreated with or without the indicated reagents for 30 min. Zeatin riboside or CGS (0.1 µM) was added for another 24 h. Cell viability was expressed as a percentage of the results from the MTT and trypan blue exclusion assays with respect to the mean value of the serum-contained control group. Data points represent the mean ± SEM (*n* = 3∼6). *p<0.05, compared to its serum-deprived group. (C) Serum-deprived cells were pretreated with or without 1 µM ZM or 1 µM SCH for 30 min. Zeatin riboside was added for 24 h and followed by Annexin V-FITC staining. Cells were subjected to image and cytometry analysis. Bar represents 50 µm. Data points represent the mean ± SEM (*n* = 3∼6). *p<0.05, compared to the serum-contained control group. (D) Serum-contained or -deprived cells in the presence or absence of zeatin riboside were harvested and subjected to a Western blot analysis. The relative optical density of the bands were quantified by densitometry relative to actin and normalized to the levels in serum-contained control group or in serum-deprived for 24 h group. Data points (mean ± SEM) represent one out of three independent experiments that gave similar results. *p<0.05, compared to its serum-containing control group. #*p*<0.05, compared to its 24 h serum-deprived group. AU represents arbitrary unit.

Kinetin riboside and zeatin riboside, but not kinetin, zeatin, or N^6^-benzyladenine, significantly prevented serum deprivation-induced cell death (Fig. 1A). As the positive controls, NGF (Fig. 1A) and CGS 21680 (CGS; a commercially available A_2A_-R agonist) also prevented serum deprivation-induced cell death (Fig. 1B) [30]. Since zeatin riboside at 100 µM exerted the highest protection with the exception of that by kinetin riboside, 100 µM zeatin riboside was used throughout the following experiments. Zeatin riboside also reversed H_2_O_2_–induced cell death (Fig. 1A). Pharmacologically, two commercially available A_2A_-R-specific antagonists [ZM 241385 (ZM) and SCH 58218 (SCH)] and a PKA inhibitor (H-89) dose-dependently blocked the protection by zeatin riboside (Fig. 1B). The blockade of zeatin riboside protection by ZM was re-confirmed by trypan blue exclusion assay (Fig. 1B). In addition, zeatin riboside prevented serum deprivation-induced increased fluorescence of Annexin V-FITC (a marker of apoptotic events) (Fig. 1C). As expected, ZM and SCH blocked the protective effect of zeatin riboside as revealed by imaging (Fig. 1C, upper panels) and flow cytometry studies (Fig. 1C, lower panels). Further, during the differential time course of serum deprivation, zeatin riboside significantly attenuated serum deprivation-induced cleavage of poly (ADP-ribose) polymerase (PARP) and caspase-3 at 24 h (Fig. 1D).

### Zeatin Riboside Targets the A_2A_-R and Decreases Mutant Htt Aggregations which Impair Proteasome Activity

**Figure 2:**
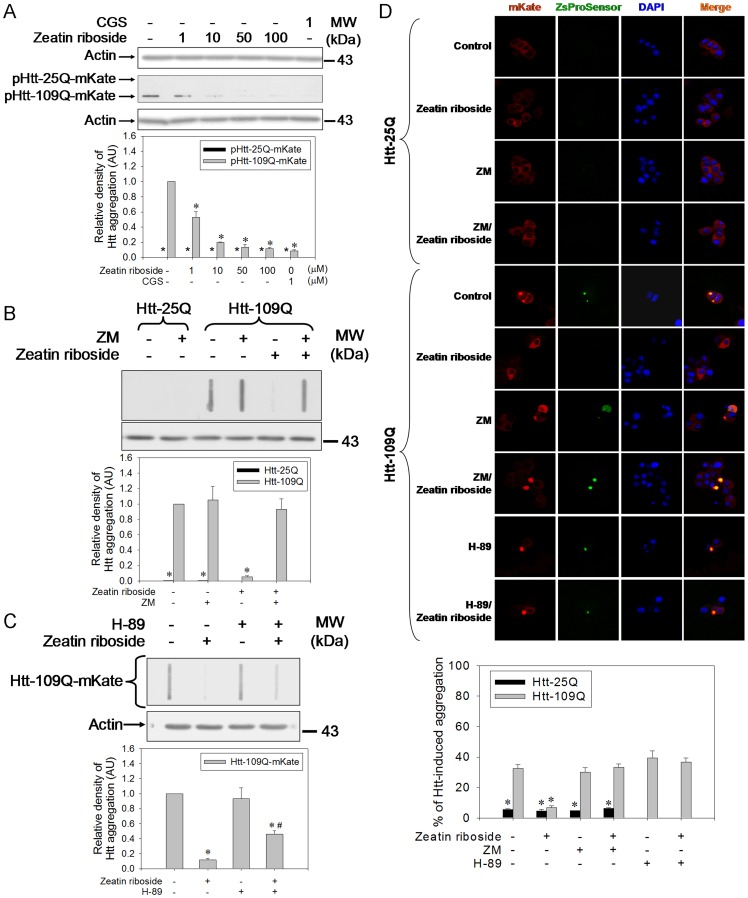
Zeatin riboside acting on the A_2A_-R attenuates mutant Htt aggregations. (A) pHtt-25Q-mKate- or pHtt-109Q-mKate-transfected cells were treated with 1 µM CGS or zeatin riboside for 24 h. Cells were harvested and subjected to a filter retardation assay and Western blot analysis. (B) The transfected cells were pretreated with or without 1 µM ZM for 30 min and treated with zeatin riboside for another 24 h. Cells were harvested and subjected to a filter retardation assay and Western blot analysis. (C) The transfected cells were pretreated with or without 5 µM H-89 for 30 min and treated with zeatin riboside for another 24 h. Cells were harvested and subjected to a filter retardation assay and Western blot analysis. The relative optical density of the bands (A∼C) were quantified by densitometry relative to actin and normalized to the levels under the Htt-109Q-overexpressed control condition. Data points represent the mean ± SEM. *p<0.05, compared to the mutant Htt control group. #*p*<0.05, compared to the zeatin riboside-treated mutant Htt group. (D) After 1 µM ZM pretreatment for 30 min, pHtt-25Q-mKate- or pHtt-109Q-mKate-transfected and pZsProSensor-co-transfected cells were treated with or without zeatin riboside for another 24 h and subjected to a confocal microscopic analysis. Bar represents 5 µm. In each group, the mKate-aggregated cells in proportion to the transfected cells were counted (100∼150 cells) These data points (mean ± SEM) represent one out of three independent experiments that gave similar results. *p<0.05, compared to the mutant Htt control group.

The filter retardation assay and confocal microscopic study revealed that zeatin riboside significantly decreased mutant Htt (109Q) aggregations (Fig. 2A–C). CGS also prevented mutant Htt aggregations (Fig. 2A). However, in the absence and presence of zeatin riboside treatment, normal Htt (25Q) failed to induce any significant aggregations (Fig. 2A–C). ZM or H-89 pretreatment significantly reversed the zeatin riboside-induced decrease in mutant Htt aggregations (Fig. 2B–D). Alternatively, as revealed by contransfection with pZsProsensor, mutant Htt, but not normal Htt, resulted in an increased intensity of green fluorescent proteins (GFPs) (Fig. 2D). Zeatin riboside significantly blocked this phenomenon, which was also reversed by ZM pretreatment (Fig. 2D).

### Inhibition of Proteasome Exacerbates Htt Aggregations

**Figure 3:**
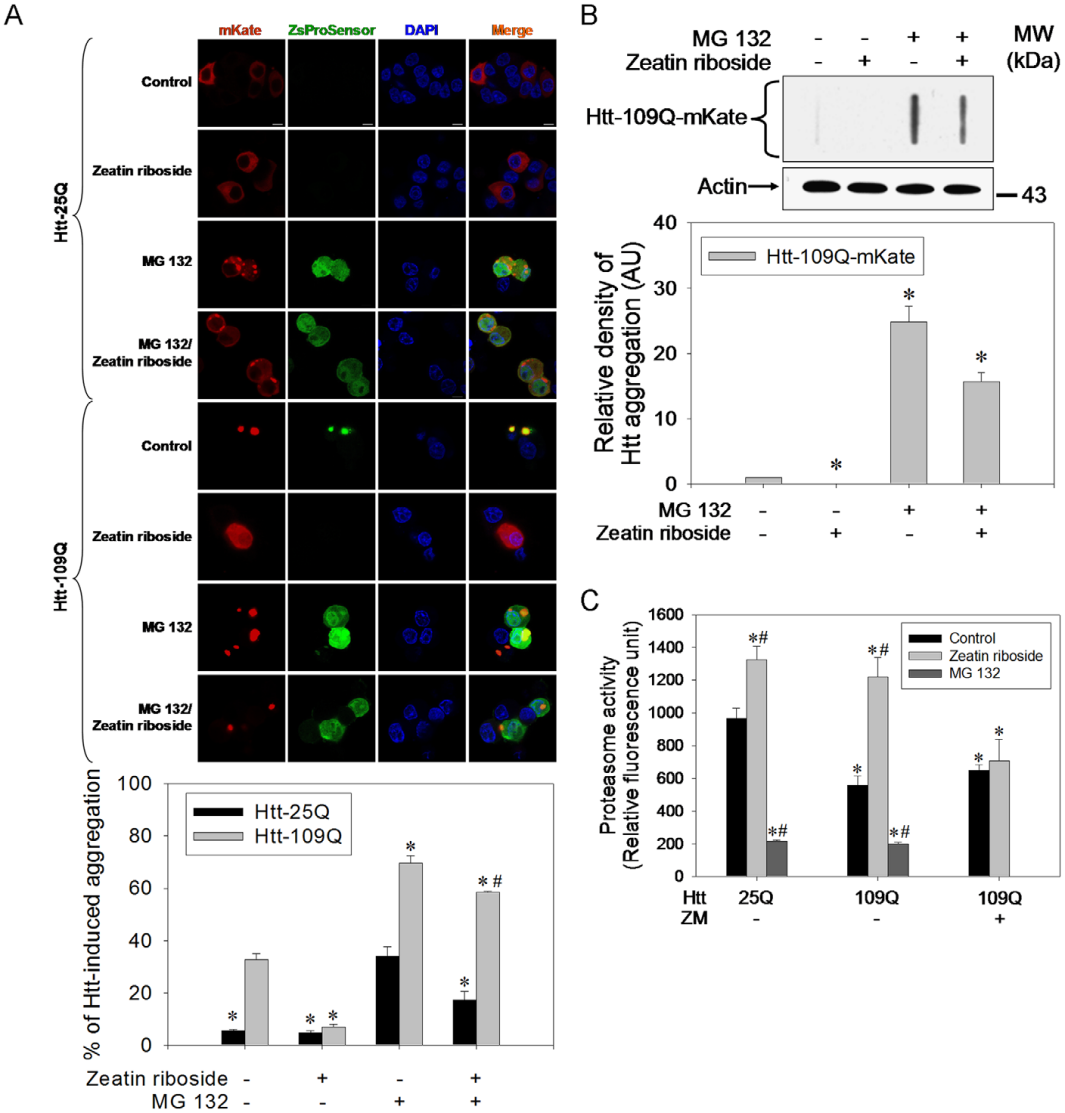
Zeatin riboside attenuates mutant Htt aggregations through increasing proteasome activity. (A) With or without 1 µM MG 132 pretreatment for 30 min and following the presence or absence of zeatin riboside for 24 h, pHtt-25Q-mKate- or pHtt-109Q-mKate-transfected and pZsProSensor-co-transfected cells were subjected to a confocal microscopic analysis. Bar represents 5 µm. In each group, the mKate-aggregated cells in proportion to the transfected cells were counted (100∼150 cells). Data points represent the mean ± SEM. *p<0.05, compared to the mutant Htt control group. #*p*<0.05, compared to the MG 132-treated mutant Htt group. (B) With or without 1 µM MG 132 pretreatment for 30 min, pHtt-109Q-mKate-transfected cells were treated with or without zeatin riboside for 24 h and subjected to a filter retardation assay and Western blot analysis. The relative optical density of the bands were quantified by densitometry relative to actin and normalized to the levels under the Htt-109Q-overexpressed control condition which was set as 1.0. Data points represent the mean ± SEM. *p<0.05, compared to the mutant Htt control group. AU represents arbitrary unit. (C) pHtt-25Q-mKate-transfected cells were treated with or without zeatin riboside or 1 µM MG 132. pHtt-109Q-mKate-transfected cells were pretreated with or without 1 µM ZM for 30 min and then supplemented with or without zeatin riboside or 1 µM MG 132 for 24 h and subjected to a proteasome activity assay. *p<0.05, compared to the Htt-25Q control group. #*p*<0.05, compared to the Htt-109Q control group. These data represent one out of three independent experiments that gave similar results.

MG 132, a proteasome inhibitor, significantly induced intense green fluorescence in normal Htt-overexpressed cells (Fig. 3A). In addition, MG 132 exacerbated mutant Htt-induced protein aggregations and reversed zeatin riboside-induced suppression of Htt aggregations (Fig. 3A and 3B). Compared to Htt-25Q overexpression, that of Htt-109Q significantly reduced proteasome activity (Fig. 3C). MG 132 drastically inhibited proteasome activity in both Htt-25Q- and Htt-109Q-overexpressing cells (Fig. 3C). ZM significantly attenuated zeatin riboside-induced increased proteasome activity in both Htt-25Q- and Htt-109Q-overexpressing cells (Fig. 3C).

### Activation of PKA Results in a Decrease in Mutant Htt Aggregations

**Figure 4:**
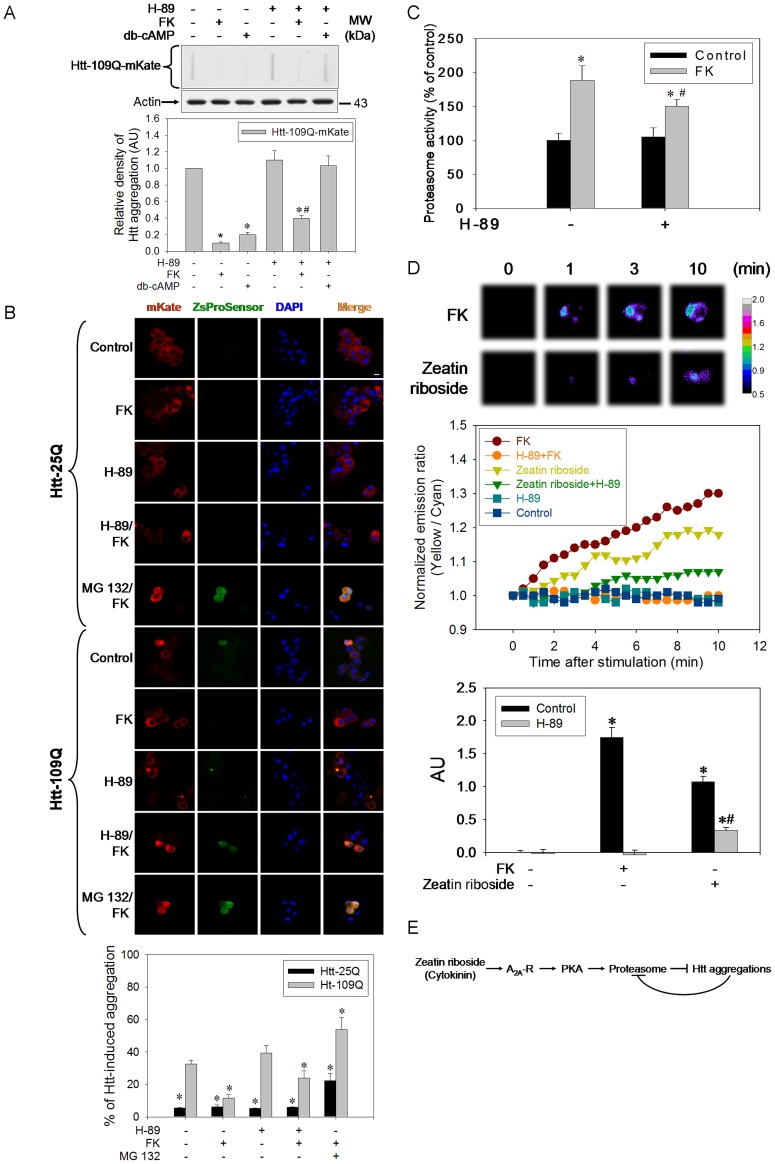
PKA attenuates mutant Htt aggregations through increasing proteasome activity. (A) With or without 1 µM H-89 pretreatment for 30 min, pHtt-109Q-mKate-transfected cells were treated with or without 10 µM FK or 100 µM db-cAMP for 24 h and subjected to a filter retardation assay and Western blot analysis. The relative optical density of the bands were quantified by densitometry relative to actin and normalized to the levels under the Htt-109Q-overexpressed control condition which was set as 1.0. Data points represent the mean ± SEM. *p<0.05, compared to the mutant Htt control group. #*p*<0.05, compared to the FK-treated mutant Htt group. AU represents arbitrary unit. (B) After 5 µM H-89 or 1 µM MG 132 pretreatment for 30 min, pHtt-25Q-mKate- or pHtt-109Q-mKate-transfected and pZsProSensor-co-transfected cells were treated with or without 10 µM FK for 24 h and subjected to a confocal microscopic analysis. In each group, the mKate-aggregated cells in proportion to the transfected cells were counted (100∼150 cells). Data points represent the mean ± SEM. *p<0.05, compared to the mutant Htt control group. (C) With or without 5 µM H-89 pretreatment for 30 min, pHtt-109Q-mKate-transfected cells were supplemented with or without zeatin riboside or 10 µM FK for 24 h and subjected to a proteasome activity assay. *p<0.05, compared to the control group. ^#^ *p*<0.05, compared to the FK-treated but without H-89-pretreated group. (D) With or without 10 µM H-89 pretreatment for 30 min, AKAR1-transfected cells were added with or without 10 µM FK or zeatin riboside and the FRET images (upper panel) were acquired and analyzed (middle panel). Bar represents 5 µm. In each group, the area under the curve (AUC) subtracting with the background level was calculated and plotted in arbitrary unit (AU) (lower panel). Data represents the mean ± SEM (*n* = 3∼6). *p<0.05, compared to the control group. (E) Zeatin riboside-mediated suppression of mutant Htt aggregations involves activation of the adenosine A_2A_ receptor (A_2A_-R), PKA, and proteasome. Mutant Htt aggregations in turn inhibit proteasome activity. These data represent one out of three independent experiments that gave similar results.

Forskolin (FK) and dibutyl-cyclic AMP (db-cAMP) (two known PKA activators) significantly attenuated mutant Htt-induced protein aggregations, while H-89 reversed this phenomenon (Fig. 4A, B). In addition, FK also blocked mutant Htt-induced increases in GFPs (Fig. 4B). H-89 blocked this phenomenon afforded by FK (Fig. 4B). MG 132 not only blocked the protection by FK but also exacerbated Htt aggregations in both 25Q- and 109Q-overexpressing cells (Fig. 4B). FK had no effect on normal Htt or GFP expressions (Fig. 4B). FK significantly increased proteasome activity in both normal and mutant Htt-overexpressed cells; however, H-89 pretreatment blocked the effects of FK (Fig. 4C). Additionally, overexpression of mutant Htt decreased proteasome activity (Fig. 4C). In addition, FK and zeatin riboside significantly increased the ratio of YFP/CFP in AKAR1-transfected cells; however, H-89 pretreatment blocked the effects of both FK and zeatin riboside (Fig. 4D).

## Discussion

### Zeatin Riboside Mediates the A_2A_-R Signalings and Prevents Serum Deprivation-induced Apoptosis

According to our recently published pharmacophore models of the A_2A_-R [31], the K*i* of some cytokinins were ranged from 2.9 to 46 µM (Supplement S1). We next tested their biological functions using a serum deprivation-induced cell death model that highlighted the functional role of the A_2A_-R [30]. Among these cytokinins, only kinetin and zeatin with riboside were protective in this model (Fig. 1A). The inabilities of other cytokinins in preventing cell death were not clear. Further works were required to be done to reveal if they could act as antagonists. The protection by zeatin riboside was blocked by two A_2A_-R antagonists, ZM and SCH (Fig. 1B), indicating an A_2A_-R-mediated effect. The protections of NGF and CGS (Fig. 1A–B) were also consisted with the previously published article [30]. Besides, except serum deprivation-induced cell death, zeatin riboside also prevented H_2_O_2_-induced cell death (Fig. 1A), further confirming the protections of zeatin riboside. Annexin V-FITC staining confirmed that ZM and SCH could block the protection by zeatin riboside in antagonizing serum deprivation-induced apoptosis (Fig. 1C). A time course study of serum deprivation-induced cleavage of PARP and caspase 3 which was blocked by zeatin riboside (Fig. 1D) also confirmed that serum deprivation induces apoptosis [30]. Furthermore, the above identified protectants (CGS and NGF) and other known protectants (db-cAMP and FK) [30] were also confirmed to prevent serum deprivation-induced cleavage of PARP (Supplement S2). Although serum deprivation-induced apoptosis is well documented, there are still different forms of death events should be concerned, for instance, serum deprivation-induced autophagic cell death [32], [33] or a new form of death (parthanatos) which is triggered by the nuclear translocation of mitochondrial apoptosis inducing factor resulting in caspase-independent cell death [34]. Thus, the involvement of zeatin riboside in antagonizing other form of cell death is currently unknown and requires further investigation.

Normally, signaling of the A_2A_-R sequentially couples with G*_S_α* and involves activation of adenylyl cyclase, formation of cAMP, stimulation of PKA, and phosphorylation activation of the CREB at Ser133 [30], [35], [36], which plays a pivotal role in neuronal survival [37] and genetic models of HD [38]. We further examined and found that zeatin riboside did induce CREB phosphorylation which could also be blocked by ZM and H-89 (Supplement S3A). Besides, CREB overexpression blocked mutant Htt aggregations (Supplement S3B) may further confirm the importance of A_2A_-R signalings in this system. Therefore, since the A_2A_-R has implicated as a therapeutic target in treating HD [25], [26], [39], we thus tested if zeatin riboside could be a candidate to treat HD.

### Zeatin Riboside Targets the A_2A_-R and Decreases Mutant Htt Aggregations that Impair Proteasome Activity

In order to study HD outside of animal models, several cell models were established and examined [40], [41]. In this study, plasmid which harbors the exon 1 region of the Htt gene with 25 or 109 CAG repeats conjugating with the red fluorescent protein (mKate) as a reporter were transiently transfected (Supplement S4). The filter assay revealed that zeatin riboside dose-dependently attenuated mutant Htt aggregations (Fig. 2A). CGS also prevented Htt aggregates in this system (Fig. 2A), consistent with previous findings [26]. Further, ZM (Fig 2B) and H-89 (Fig. 2C) attenuated zeatin riboside-induced suppression of mutant Htt aggregations, demonstrating that zeatin riboside targets the A_2A_-R and the subsequent PKA to prevent mutant Htt aggregations. Alternatively, the imaging study revealed that 109Q, not 25Q, induced highly condensed and punctuated red and green fluorescent proteins (Fig. 2D), indicating decreased proteasome activity. However, zeatin riboside alleviated all of these phenomena which could be reversed by ZM or H-89 pretreatment (Fig.2D), consistent with the filter assay data. These data indicated that zeatin riboside by acting on the A_2A_-R prevents Htt aggregates and Htt aggregates impair proteasome activity [42], [43]. Therefore, it is possible that zeatin riboside could elevate proteasome activity to degrade Htt aggregates.

### Zeatin Riboside Elevates Proteasome Activity and Proteasome Inhibition Exacerbates Htt Aggregations

The ubiquitin-proteasome system (UPS) plays an essential role in degrading misfolded and damaged proteins that are polyubiquitinated by ubiquitin ligases and targeted to proteasomes for degradation, such as mutant Htt [44]. We thus examined the importance of proteasomes in degrading Htt aggregates. MG 132, a proteasome inhibitor, was applied, and found that MG 132 exacerbated Htt aggregations with both 109Q and 25Q (Fig. 3A), consistent with previous findings [45]. MG 132 also blocked zeatin riboside-induced suppression of mutant Htt aggregates (Fig. 3A & 3B), confirming the functional role of proteasomes in degrading Htt aggregates in this system. In addition to the imaging study, proteasome activity was also detected. Our data revealed that mutant Htt overexpression downregulated proteasome activity (Fig. 3C). However, zeatin riboside respectively elevated and reversed the proteasome activity in normal and mutant Htt-overexpressed cells. MG 132 drastically inhibited proteasome activity in normal and mutant Htt-overexpressed cells (Fig. 3C). Consequently, our data demonstrated that zeatin riboside elevated proteasome activity and then promoted mutant Htt degradation by acting on the A_2A_-R.

### PKA Activation Increases Proteasome Activity and Mutant Htt Degradation

Since PKA is the downstream target of A_2A_-R, we subsequently examined if PKA was also involved in mediating Htt aggregates in this system. Indeed, both FK- and db-cAMP-induced suppression of mutant Htt aggregates were reversed by H-89 (Fig. 4A), which indicates a positive role of PKA in suppressing mutant Htt aggregations. Alternatively, the inhibition by H-89 of FK-induced suppression of Htt aggregates (Fig. 4B) and the increase in proteasome activity (Fig. 4C) could also be reproduced using a confocal study and activity assay, further confirming PKA-mediated activation of proteasome activity and degradation of Htt aggregates. Besides, in order to demonstrate whether zeatin riboside resulted in PKA activation, a genetically encoded reporter (AKAR1) of PKA activity was used [46]. As expected, H-89 blocked both zeatin riboside- and FK-induced FRET responses, (Fig. 4D), demonstrating that zeatin riboside does mediate PKA. Therefore, zeatin riboside-induced suppression of Htt aggregations is mediated by the A_2A_-R and subsequent PKA-dependent pathways (Fig. 4E). Currently, the mechanism of mutant Htt-induced impairment of the UPS is not clear [43], [47]; although it was proposed that polyQ peptides may be transiently retained in the proteolytic core, thus impairing proteasome activity. However, it is also possible that mutant Htt aggregates are difficult to recognize by proteasomes. Further investigations are required to answer these questions.

### Implications of Cytokinins

Recently, biomedical implications of cytokinins have gradually garnered attention. For instance, kinetin was found to alleviate some messenger (m)RNA splicing diseases, such as familial dysautonomia [48]. It was also shown to delay the onset and decrease the extent of aging characteristics in cultured human skin fibroblasts [6] and is widely marketed today in a variety of skin-care products. Although kinetin was reported to protect against oxidative damage to both DNA and proteins [5], [49], the molecular mechanism for the anti-aging properties is unknown. Since kinetin is currently used only for topical applications, toxicity studies are currently underway to assess its potential as a treatment for familial dysautonomia. On the other hand, zeatin has been found to prevent scopolamine-induced memory impairment in mice [50] and β-amyloid-induced PC12 cell neurotoxicity [51], suggesting the therapeutic potential of cytokinins in treating neurodegeneration.

In this study, for the first time, we pharmacologically demonstrated that zeatin riboside can target the A_2A_-R to prevent serum deprivation-induced apoptosis and mutant Htt aggregations, suggesting a therapeutic potential in treating neuronal injury and neurodegeneration. In addition, our previously published articles show that a novel compound, N-(4-hydroxybenzyl)adenosine purified from *Gastrodia elata* [52], had A_2A_-R-binding potency that exerted protection in treating R6/2 mice (an HD animal model) [53]. This compound may be a novel therapeutic drug and lead to the development of new drugs to treat other neurodegenerative diseases [31]. Therefore, regarding plant hormones, cytokinins, the application of zeatin riboside or others with A_2A_-R-binding affinities may also be possibly implicated as novel neuroprotectants and leads for alternative treatments of neurodegeneration [25], [54].

Taken together, in this study, we showed that zeatin riboside could prevent serum deprivation-induced apoptosis and mutant Htt aggregations through activation of the A_2A_-R and subsequent PKA-dependent pathways. These findings also indicate the therapeutic potential of zeatin riboside in treating neuronal injury, HD, and other polyQ diseases, such as spinocerebellar ataxias.

## Materials and Methods

### Reagents and Cell Culture

All reagents were purchased from Sigma Chemical (St. Louis, MO, USA) except where otherwise specified. Nerve growth factor (NGF) was purchased from Alomone Labs Ltd (Jerusalem, Israel). 4-[2-[[6-Amino-9-(*N*-ethyl-β-D-ribofuranuronamidosyl)-9*H*-purin-2-yl]amino]ethyl] benzene pro-panoic acid (CGS 21680), 2-(2-furanyl)-7-(2-phenylethyl)-7*H*-pyrazolo[4,3-*e*][1], [2], [4] triazolo[1,5-*c*]pyrimidin-5-amine (SCH 58261), and 4-(2-[7-amino-2-(2-furyl)[1], [2], [4] triazolo[2,3-*a*][1], triazin-5-ylamino]ethyl)phenol (ZM 241385) were purchased from Tocris (Bristol, UK). H-89 was purchased from Biomol (Plymouth Meeting, PA, USA). All antibodies were purchased from Millipore (Bedford, MA, USA) except where otherwise specified. The anti-PARP antibody was purchase from Epitomics (1078; Burlingame, CA, USA). The anti-cleaved caspase 3 antibody was purchase from Cell Signaling (9664; Danvers, MA, USA). Plasmids including the proteasome sensor vector (pZsProSensor-1) were purchased form Clontech (Mountain View, CA, USA). Restriction enzymes were purchased from Fermantas (Vilnius, Lithuania). The AKAR1 plasmid was obtained from Dr. Roger Y. Tsien (Department of Pharmacology, Department of Chemistry & Biochemistry, University of California, San Diego, CA, USA). Dulbecco's modified Eagle's medium (DMEM), fetal bovine serum (FBS), and horse serum were purchased from HyClone (Logan, UT, USA). Rat PC12 cells purchased from American Type Culture Collection (ATCC; Manassas, VA, USA) were maintained in DMEM supplemented with 10% horse serum and 5% FBS and incubated in a CO_2_ incubator (5%) at 37°C. pHtt-25Q-mKate and pHtt-109Q-mKate were prepared as described previously [55].

### MTT and Trypan Blue Exclusion Assays

Survival was assessed by the 3-(4,5-dimethylthiazol-2-yl)-2,5-diphenyl tetrazolium bromide (MTT) metabolism assay as described previously [56]. In brief, after treatment, MTT was added to the medium (0.5 mg/ml) and incubated at 37°C for 2∼3 h. The plating number was 10^4^ cells/well in a 96-well plate. After discarding the medium, DMSO was applied to the well to dissolve the formazan crystals, and the absorbances at 570 and 630 nm in each well were measured on a micro-enzyme-linked immunosorbent assay (ELISA) reader. In addition, after different treatments, cells growing on 6-well (4×10^5^ cells/well) plate were scraped and counted using a hemacytometer after trypan blue staining (0.3%).

### Transient Transfection

Lipofectamine™ 2000 (Invitrogen) was used as a vehicle to transfer plasmids into cells as described by the protocol. Normally, 5 µg of DNA combined with 5 µl of Lipofectamine™ 2000 was applied to each well of 6-well plates. The plating number was (1∼1.5)×10^6^ cells/well. After transfections for 6 h, cells were treated with reagents for another 24 h. Images were then taken with a Zeiss Axiovert 200 M inverted fluorescence microscope (Göttingen, Germany).

### Annexin V-FITC Staining and Analysis

An Annexin V (FITC-conjugated) apoptosis kit (K101–400; BioVision, Mountain View, CA, USA) was used to analyze apoptotic cells. The experimental protocol followed the manufacturer's instructions. In brief, after a 24-h treatment, cells growing on 12-well plates at (3∼4)×10^5^ cells/well were loaded with 0.5 ml binding buffer and 5 µl Annexin V-FITC. After incubation for 5 min in the dark, cells were washed once with 1 ml of culture medium (without phenol red) to take phase contrast and fluorescent micrographs. Besides, the stained-cells could also be used for flow cytometry analysis (FACScan®, Becton Dickinson, Franklin Lakes, NJ). The mean values of the fluorescent intensities of FITC were collected using an FL-1 channel (488/530^Ex/Em^ nm). Five thousand live cells were analyzed per sample.

### Western Blot Analysis

Equal amounts of cell lysates (20 µg/well) derived from the filtered assay were separated by SDS-polyacrylamide gel electrophoresis (PAGE) and then electroblotted onto Immobilon polyvinylidene difluoride (PVDF) membranes (Millipore). Membranes were blocked with 5% skim milk in TBST (100 mM Tris-HC1 and 150 mM NaC1; pH 7.4, containing 0.05% Tween 20) for 1 h at room temperature and then incubated with the first antibody (1/1000∼2000) at 4°C for overnight. The anti-PARP antibody was used to probe pro- and cleaved-form of PARP. The anti-caspase 3 (9662; Cell Signaling, Danvers, MA, USA) and anti-cleaved caspase 3 antibodies were respectively used to probe pro- and cleaved-form of caspase 3. Actin was used as an internal control and probed with anti-actin (MAB1501) antibody. After three washes with TBST, the blot was incubated with a second antibody (1∶5000) conjugated to horseradish peroxidase for 1 h, processed for visualization using an enhanced chemiluminescence system (Pierce, Rockford, IL, USA), and exposed to Kodak XAR-5 film (Rochester, New York, USA) to obtain the fluorographic images. The freeware ImageJ (http://imagej.nih.gov/ij/download.html) was used to measure the required image density.

### Filter Retardation Assay

This method followed that described by Wanker et al. [57] with a few modifications. In brief, harvested cells were resuspended in lysis buffer (50 mM Tris-HCl (pH 8.8), 100 mM NaCl, 5.0 mM MgCl_2_, 1 mM EDTA, and 0.5% (w/v) IPGEAL containing 1× protease inhibitor cocktail (Roche Diagnostics, Indianapolis, IN, USA)) and sonicated for 10 s (1 pulse/s). Equal protein concentrations (15∼20 µg/well) in each group were filtered through a 2% sodium dodecylsulfate (SDS)-pre-equilibrated cellulose-acetate membrane (0.2 µm; Whatman, Maidstone, Kent, UK) using the Bio-Dot SF Apparatus (Bio-Rad, Hercules, CA, USA). During suction, each well was washed with 200 µl 0.1% SDS twice. The blot was blocked in TBS (100 mM Tris-HC1 and 150 mM NaC1; pH 7.4) containing 3% nonfat dried milk for 1 h at room temperature and then incubated with the anti-polyglutamine (1∶5000; MAB1574) antibody in 3% bovine serum albumin (BSA) with 0.02% NaN_3_ (4°C overnight) to probe normal and mutant Htts. The subsequent methods were the same as those described above.

### Proteasome Activity Assay

Proteasome activity was indirectly studied by transfecting pZsProSensor-1 (Clontech), a eukaryotic expression vector designed to express ZsGreen fused to the mouse ornithine decarboxylase degradation domain which is highly susceptible to proteasome degradation. Therefore, this vector was used to monitor proteasome activity in living cells. Normally, if proteasomes are active in living cells, the protein does not accumulate. However, when proteasome activity decreases, such as the addition of a proteasome inhibitor, the fusion protein accumulates in cells resulting in increased green fluorescence. Therefore, proteasome activity is inversely correlated with the green fluorescence. Alternatively, a 20S Proteasome Activity Assay kit (APT280; Millipore, Billerica, MA, USA) was used to measure the proteasome activity according to the protocol.

### PKA Activity Assay

This method was carried out as described by Zhang et al. [46]. In brief, after AKAR1 transfection for 24 h, cells were imaged on a Zeiss Axiovert 200 M microscope with a 40×/1.3NA oil-immersion objective lens and a cooled CCD camera (CoolSNAP HQ^2^; Photometrics, Tucson, AZ, USA). Dual-emission ratio imaging was acquired with a 420DF20 excitation filter, a 450DRLP dichroic mirror, and two emission filters (475DF40 for cyan and 535DF25 for yellow (Chroma Technology, Bellows Falls, VT, USA)) altered by a filter changer (Lambda DG-4; Sutter Instruments, San Rafael, CA, USA). Fluorescence images were background-corrected. Exposure times were 50∼200 ms, and images were taken every 30∼60 s.

### Confocal Microscopy

Transfected cells growing on poly-L-lysine-coated cover slides were fixed with 4% paraformaldehyde at room temperature for 10 min, and DAPI (1 µg/ml) stain was applied for another 10 min. After three washes with PBS, cover slides were mounted onto glass slides with Aqua Poly-Mount (Polysciences, Warrington, PA, USA). Images of cells were visualized with a Leica TSC SP confocal laser scanning microscope (Wetzlar, Germany).

### Statistical Analysis

Results were analyzed by one- or two-way analysis of variance (ANOVA) according to which was appropriate. Two-way ANOVA with repeated measurements were used to analyze the differences in PKA activity assay. Differences between means were assessed by the Student-Newman-Keuls method and were considered significant at p<0.05.

## Supporting Information

Supplement S1 **The Ki of cytokinin on molecular modeling of A_2A_-R.** Purple and cyan circles respectively represent hydrogen and bond donor.
(DOC)

Supplement S2 **CGS, db-cAMP, FK, and NGF prevent serum deprivation-induced PARP cleavage.** Serum-contained or -deprived cells in the presence or absence of CGS (0.1 µM), db-cAMP (100 µM), FK (10 µM), or NGF (50 ng/ml) were harvested and subjected to the Western blot analysis.
(DOC)

Supplement S3 **Zeatin riboside activates the cAMP response element-binding protein (CREB) through a protein kinase A (PKA)-dependent pathway.** (A) Cells deprived of serum were pretreated with 5 µM H-89 or 1 µM ZM for 30 min and then treated in the presence or absence of zeatin riboside or 10 µM FK for 1 h. Cells were harvested and subjected to a Western blot analysis. (B) Cells transfected with pHtt-109Q-mKate were also co-transfected with or without pCMV-CREB or promoter-less pEGFP for 24 h. Cells were harvested and subjected to the filter retardation assay and Western blot analysis.
(DOC)

Supplement S4 **Zeatin riboside prevents mutant Htt (109Q)-induced aggregations.** (A) After pretreatment with ZM or H-89 for 30 min, cells over-expressing normal Htt-25Q-mKate and mutant Htt-109Q-mKate were treated with or without 100 µM zeatin riboside for 24 h. The images of cells in red fluorescence (mKate) and bright field (BF) were taken by a fluorescence microscope. Bar represents 50 µm.
(DOC)
