# Supplementary material for: When Cytokinin, a Plant Hormone, Meets the Adenosine A2A Receptor: A Novel Neuroprotectant and Lead for Treating Neurodegenerative Disorders?
Source: PLoS One. 2012 Jun 18;7(6):e38865. doi: 10.1371/journal.pone.0038865 (PMC3377719; doi:10.1371/journal.pone.0038865)
Supplement: Supplement S1 — The Ki of cytokinin on molecular modeling of A2A-R. Purple and cyan circles respectively represent hydrogen and bond donor. (DOC) [file pone.0038865.s001.doc]

**Supplement S1: The Ki of cytokinin on molecular modeling of A_2A_-R. Purple and cyan circles respectively represent hydrogen and bond donor.**
