# Supplementary material for: When Cytokinin, a Plant Hormone, Meets the Adenosine A2A Receptor: A Novel Neuroprotectant and Lead for Treating Neurodegenerative Disorders?
Source: PLoS One. 2012 Jun 18;7(6):e38865. doi: 10.1371/journal.pone.0038865 (PMC3377719; doi:10.1371/journal.pone.0038865)
Supplement: Supplement S2 — CGS, db-cAMP, FK, and NGF prevent serum deprivation-induced PARP cleavage. Serum-contained or -deprived cells in the presence or absence of CGS (0.1 µM), db-cAMP (100 µM), FK (10 µM), or NGF (50 ng/ml) were harvested and subjected to the Western blot analysis. (DOC) [file pone.0038865.s002.doc]

**Supplement S2: NGF, FK, db-cAMP, and CGS prevent serum deprivation-induced PARP cleavage.** Serum-contained or -deprived cells in the presence or absence of CGS (0.1 μM), db-cAMP (100 μM), FK (10 μM), or NGF (50 ng/ml) were harvested and subjected to the Western blot analysis.
