# Supplementary material for: When Cytokinin, a Plant Hormone, Meets the Adenosine A2A Receptor: A Novel Neuroprotectant and Lead for Treating Neurodegenerative Disorders?
Source: PLoS One. 2012 Jun 18;7(6):e38865. doi: 10.1371/journal.pone.0038865 (PMC3377719; doi:10.1371/journal.pone.0038865)
Supplement: Supplement S3 — Zeatin riboside activates the cAMP response element-binding protein (CREB) through a protein kinase A (PKA)-dependent pathway. (A) Cells deprived of serum were pretreated with 5 µM H-89 or 1 µM ZM for 30 min and then treated in the presence or absence of zeatin riboside or 10 µM FK for 1 h. Cells were harvested and subjected to a Western blot analysis. (B) Cells transfected with pHtt-109Q-mKate were also co-transfected with or without pCMV-CREB or promoter-less pEGFP for 24 h. Cells were harvested and subjected to the filter retardation assay and Western blot analysis. (DOC) [file pone.0038865.s003.doc]

**Supplement S3: Zeatin riboside activates the cAMP response element-binding protein (CREB) through a protein kinase A (PKA)-dependent pathway.** (A) Cells deprived of serum were pretreated with 5 μM H-89 or 1 μM ZM for 30 min and then treated in the presence or absence of zeatin riboside or 10 μM FK for 1 h. Cells were harvested and subjected to a Western blot analysis. (B) Cells transfected with pHtt-109Q-mKate were also co-transfected with or without pCMV-CREB or promoter-less pEGFP for 24h. Cells were harvested and subjected to the filter retardation assay and Western blot analysis.

A

B
