# Supplementary material for: When Cytokinin, a Plant Hormone, Meets the Adenosine A2A Receptor: A Novel Neuroprotectant and Lead for Treating Neurodegenerative Disorders?
Source: PLoS One. 2012 Jun 18;7(6):e38865. doi: 10.1371/journal.pone.0038865 (PMC3377719; doi:10.1371/journal.pone.0038865)
Supplement: Supplement S4 — Zeatin riboside prevents mutant Htt (109Q)-induced aggregations. (A) After pretreatment with ZM or H-89 for 30 min, cells over-expressing normal Htt-25Q-mKate and mutant Htt-109Q-mKate were treated with or without 100 µM zeatin riboside for 24 h. The images of cells in red fluorescence (mKate) and bright field (BF) were taken by a fluorescence microscope. Bar represents 50 µm. (DOC) [file pone.0038865.s004.doc]

**Supplement S4: Zeatin riboside prevents mutant Htt (109Q)-induced aggregations.** After pretreatment with ZM or H-89 for 30 min, cells over-expressing normal Htt-25Q-mKate and mutant Htt-109Q-mKate were treated with or without 100 μM zeatin riboside for 24h. The images of cells in red fluorescence (mKate) and bright field (BF) were taken by a fluorescence microscope. Bar represents 50 μm.
